# Psychological Problems among Head and Neck Cancer Patients in Relation to Utilization of Healthcare and Informal Care and Costs in the First Two Years after Diagnosis

**DOI:** 10.3390/curroncol29050260

**Published:** 2022-04-30

**Authors:** Florie E. van Beek, Femke Jansen, Rob J. Baatenburg de Jong, Johannes A. Langendijk, C. René Leemans, Johannes H. Smit, Robert P. Takes, Chris H. J. Terhaard, José A. E. Custers, Judith B. Prins, Birgit I. Lissenberg-Witte, Irma M. Verdonck-de Leeuw

**Affiliations:** 1 Department of Clinical, Neuro- and Developmental Psychology, Faculty of Behavioral and Movement Sciences, Amsterdam Public Health Research Institute, Vrije Universiteit Amsterdam, van der Boechorststraat 1, 1081 BT Amsterdam, The Netherlands; f.e.vanbeek@amsterdamumc.nl; 2 Department of Otolaryngology-Head and Neck Surgery, Amsterdam Public Health Research Institute, Amsterdam UMC Location VUmc, De Boelelaan 1117, 1081 HV Amsterdam, The Netherlands; cr.leemans@amsterdamumc.nl (C.R.L.); im.verdonck@amsterdamumc.nl (I.M.V.-d.L.); 3 Department of Otolaryngology and Head and Neck Surgery, Erasmus Cancer Institute, Erasmus MC, 3015 GD Rotterdam, The Netherlands; r.j.baatenburgdejong@erasmusmc.nl; 4 Department of Radiation Oncology, University Medical Center Groningen, University of Groningen, 9713 GZ Groningen, The Netherlands; j.a.langendijk@umcg.nl; 5 Department of Psychiatry, Neuroscience Campus Amsterdam and Amsterdam Public Health Research Institute, Amsterdam UMC, VU University Medical Center, 1081 HV Amsterdam, The Netherlands; jh.smit@amsterdamumc.nl; 6 Department of Otolaryngology-Head and Neck Surgery, Radboud University Medical Center, 6525 GA Nijmegen, The Netherlands; robert.takes@radboudumc.nl; 7 Department of Radiation Oncology, University Medical Center, 3584 CX Utrecht, The Netherlands; c.h.j.terhaard@umcutrecht.nl; 8 Department of Medical Psychology 926, Radboud University Medical Center, Radboud Institute for Health Sciences, P.O. Box 9101, 6500 HB Nijmegen, The Netherlands; jose.custers@radboudumc.nl (J.A.E.C.); judith.prins@radboudumc.nl (J.B.P.); 9 Department of Epidemiology and Data Science, Amsterdam UMC, De Boelelaan 1117, 1081 HV Amsterdam, The Netherlands; b.lissenberg@amsterdamumc.nl

**Keywords:** psychology, mental health, healthcare use, costs, informal care, head and neck cancer

## Abstract

Background: To investigate associations between psychological problems and the use of healthcare and informal care and total costs among head and neck cancer (HNC) patients. Method: Data were used of the NETherlands QUality of Life and Biomedical Cohort study. Anxiety and depression disorder (diagnostic interview), distress, symptoms of anxiety and depression (HADS), and fear of cancer recurrence (FCR) and cancer worry scale (CWS) were measured at baseline and at 12-month follow-up. Care use and costs (questionnaire) were measured at baseline, 3-, 6-, 12-, and 24-month follow-up. Associations between psychological problems and care use/costs were investigated using logistic and multiple regression analyses. Results: Data of 558 patients were used. Distress, symptoms of anxiety or depression, FCR, and/or anxiety disorder at baseline were significantly associated with higher use of primary care, supportive care, and/or informal care (odds ratios (ORs) between 1.55 and 4.76). Symptoms of anxiety, FCR, and/or depression disorder at 12-month follow-up were significantly associated with use of primary care, supportive care, and/or informal care (ORs between 1.74 and 6.42). Distress, symptoms of anxiety, and FCR at baseline were associated with higher total costs. Discussion: HNC patients with psychological problems make more use of healthcare and informal care and have higher costs. This is not the result of worse clinical outcomes.

## 1. Introduction

Head and neck cancer (HNC) patients are prone to psychological problems. In this exploratory study, psychological problems are defined as symptoms of depression, symptoms of anxiety, distress, fear of cancer recurrence (FCR), depression disorder, or anxiety disorder. Prevalence rates of distress range from 29% to 53% [1,2,3]. It is estimated that symptoms of anxiety are present in approximately 10% to 29% [4,5], and symptoms of depression in 15% to 50%, of HNC patients [5,6,7,8,9,10]. Prevalence rate of high FCR is estimated at 53% among HNC patients [11]. These psychological problems, besides influencing a patients’ health-related quality of life [12], may also have economic consequences due to higher healthcare use [13].

Carlson and Bultz [13] previously suggested that cancer patients with psychological problems not only have increased mental healthcare use, but may also make more use of other healthcare domains, such as inpatient healthcare and general practitioner visits. A recent systematic review investigating psychological problems in relation to healthcare and societal costs among cancer patients in general, supported this suggestion [14]. This review showed that there is strong evidence for a significant association between anxiety and depression disorders and increased inpatient and outpatient healthcare use. When focusing on psychological symptoms (rather than disorders), FCR was found to be significantly associated with increased primary care use [15]. For other associations between psychological symptoms (FCR, distress, symptoms of depression, and symptoms of anxiety) and healthcare use (inpatient, primary, mental, and supportive care) the results were inconclusive due to inconsistent findings or limited evidence available [14]. No studies were conducted that investigated the association between psychological problems and informal care use. This review also showed that most previous research was conducted among breast cancer patients and cancer patients in general, and only a few studies targeted other specific tumor sites, such as HNC. Existing evidence on the associations between psychological problems and healthcare use may not be representative of HNC patients, as differences in sociodemographic and clinical characteristics, and differences in the prevalence of psychological problems, may influence this association. Furthermore, as studies have shown that HNC patients report one of the highest prevalences of any mental disorder in a life time and currently among all cancer types [10,16], investigating the relationship between healthcare use and costs among this cancer patient subgroup is especially important. So far, only two studies have investigated the association between psychological problems and medical healthcare use among HNC patients [17,18]. A cross-sectional study by Laurence et al. [18] found that, among 34,153 HNC patients, depression disorder was associated with more hospital admissions. Another cross-sectional study by Jeffery et al. found that, among 2944 HNC patients, depression and anxiety disorders were associated with more hospital admissions, ambulatory visits, and the number of bed days in hospital [17]. However, there are no longitudinal studies to date that investigate the association between psychological problems and mental healthcare, primary care, supportive care, and/or informal care among HNC patients. 

This exploratory study aimed to investigate the relationship between psychological problems in relation to healthcare utilization (mental healthcare, primary care, and supportive care), use of informal care, and the costs, from baseline (before the start of treatment) up to 2 years after treatment, among HNC patients.

## 2. Materials & Methods

### 2.1. Patients and Procedure

Data from the NETherlands QUality of Life and Biomedical Cohort (NET-QUBIC), an ongoing prospective observational cohort study among newly diagnosed HNC patients in the Netherlands, were used [19]. Patients were recruited between March 2014 and June 2018. Patients were included in NET-QUBIC if they were (1) 18 years or older; (2) treated with curative intent for cancer of the oral cavity, oropharynx, hypopharynx, larynx, or unknown primary; (3) able to write, read, and speak Dutch; and (4) if they completed the medical consumption questionnaire (iMCQ) at baseline. Exclusion criteria were severe psychiatric comorbidities (schizophrenia, Korsakoff’s syndrome, severe dementia). Consent procedures were approved by the Medical Ethical Committee of VUmc and followed the Dutch Medical Research Involving Human Subjects Act (METc VUmc 2013.301). The NET-QUBIC Data Warehouse comprises data derived from an electronic clinical report form (eCRF) (assessed at baseline, 24-, and 60-month follow-up); patient-reported outcome measures (PROMs); at baseline (shortly after diagnosis and before start of treatment); at 3-, 6-, 12-, 24-, 36-, 48-, and 60-month follow-up (after finishing cancer treatment); and fieldwork assessments (at baseline, 6-, 12-, 24-, and 60-month follow-up). In this study, baseline eCRF data were used, as well as PROM data collected at baseline and at 3-, 6-, 12-, and 24-month follow-up. From the fieldwork assessments, we used data from the psychiatric interview (Composite International Diagnostic Interview (CIDI)) collected at baseline and at 12-month follow-up. 

### 2.2. Outcome Measures

Demographic and clinical characteristics were collected by PROMs and eCRF data. Demographic factors included sex, age, education (low/middle/high), and living status (alone/cohabiting). Clinical factors included tumor location (oral cavity/oropharynx, hypopharynx, larynx), tumor stage (0–II/III–IV), treatment modality (single/multimodality treatment), World Health Organization performance status (0, able to carry out all normal activity without restriction; ≥1, restricted in normal activities). Comorbidity was assessed by the 27-item Adult Comorbidity Evaluation-27 Index, which categorizes comorbidity as none–mild, and moderate–severe [20]. 

Symptoms of anxiety, depression, and distress were measured with the Hospital Anxiety and Depression Scale (HADS). The HADS is a 14-item questionnaire measuring symptoms of anxiety (subscale HADS-A) and depression (subscale HADS-D) [21]. Patients respond to all items on a 4-point Likert scale, resulting in a subscale score ranging from 0 to 21. A higher score indicates higher extent of depression or anxiety symptoms. A subscale score of ≥8 was used to identify patients with symptoms of anxiety or depression. A total score of ≥11 was used to identify patients with distress. Internal consistency in this study was good (Cronbach’s alpha ranged from 0.78 to 0.89).

Fear of cancer recurrence was measured with the Cancer Worry Scale (CWS) (22). The CWS is an 8-item questionnaire measuring concerns about developing cancer or developing cancer again, and the effect of these concerns on daily life. Patients respond to all items on a 4-point Likert scale, resulting in a subscale score ranging from 8 to 32. A higher score indicates higher extent of FCR. A cut-off at ≥14 for the total score was used to identify patients with a high level of FCR [22]. The Dutch version of CWS is validated in various cancer populations [23,24]. Internal consistency was good in this HNC study population (Cronbach’s alpha was 0.89). Anxiety disorder and depression disorder in the past 6 (baseline) or 12 (12-month follow-up) months was assessed with the Composite International Diagnostic Interview (CIDI), which is based on DSM-IV criteria [25]. Fieldworkers from different backgrounds (e.g., nurse, dietician, psychologist) were trained to conduct the CIDI in a standardized way. All CIDI interviews were audiotaped and randomly checked for their quality. Healthcare use was measured with the iMCQ developed by the Institute for Medical Technology Assessment (iMTA) of the Erasmus University Rotterdam, the Netherlands [26,27]. This questionnaire measures healthcare use with a recall period of 3 months. In this study, we specifically investigated the use of (1) mental healthcare (psychiatrist, psychologist, or psychotherapist visits); (2) primary care (general practitioner visits and phone calls; (3) supportive care (physiotherapy, speech therapy, oral hygiene care, dietetics, social work, support groups); and (4) informal care (support from family, friends, neighbors, colleagues). In cases where data on the number of visits were missing (e.g., a patient reported to have visited a general practitioner, but did not report the number of visits), assumptions were made based on the means of participants who used this type of care, per measurement.

Total costs (mental healthcare, primary care, supportive care, and informal care costs) were calculated by multiplying resource use by the integral cost price from a Dutch cost price manual [28]. All prices were converted to 2018 prices using the consumer price index.

### 2.3. Statistical Analysis

Baseline characteristics of the study population are described using their mean and standard deviation, and percentage. Differences between included and excluded patients were investigated using independent *t*-tests for continuous variables and chi-square tests for categorical variables. A *p*-value lower than 0.05 was considered statistically significant. Associations between psychological problems and healthcare use (yes/no) were analyzed using chi-square tests (univariate analyses) and logistic regression analyses (multivariate analyses). Scores on symptoms of anxiety and depression, distress, and fear of recurrence were dichotomized based on validated cut-off scores, as described above. The potential confounding role of age, sex, living status, education level, tumor site, tumor stage, treatment, performance status, and comorbidity were investigated using forward logistic regression analyses. Only potential confounding factors that were significantly associated with healthcare use (*p*-value for entry of <0.05) were included in the final multivariate model. Odds ratios (OR) were calculated as a measure of effect size, and represent the increased odds for care use in HNC patients with psychological problems compared to those without. 

The association between psychological problems and healthcare costs was analyzed using multiple regression analyses corrected for all above-mentioned variables. Since cost data are usually characterized by a non-normal distribution and high variance, studies are seldom powered to detect significant differences in costs among groups [29]. Therefore, a probabilistic approach was used. Bias-corrected and accelerated bootstrap confidence intervals (BCa CI) were generated by replicating the regression analyses using bias-corrected and accelerated bootstrapping with 5000 replications.

Analyses were carried out investigating psychological problems before treatment in relation to care use and costs at baseline, as well as at 3-, 6-, 12-, and 24-month follow-up. Furthermore, analyses were carried out investigating psychological problems at 12 months after treatment in relation to care use and costs at 12- and 24-month follow-up. All statistical analyses were conducted using the IBM Statistical Package for the Social Sciences (SPSS) version 26 (IBM Corp., Armonk, NY, USA) and R version 4.0.3 (The R Foundation for Statistical Computing, Vienna, Austria).

## 3. Results

### 3.1. Study Population

Of the 739 eligible patients, 181 patients (25%) did not fill in the iMCQ at baseline, resulting in a study population of 558 patients. Patients who were included in this specific study often lived with others, were more often diagnosed with tumor stage I or II, and often had a better WHO performance state and less comorbidity, compared to those who were not included (*p* < 0.05). 

**Figure 1:**
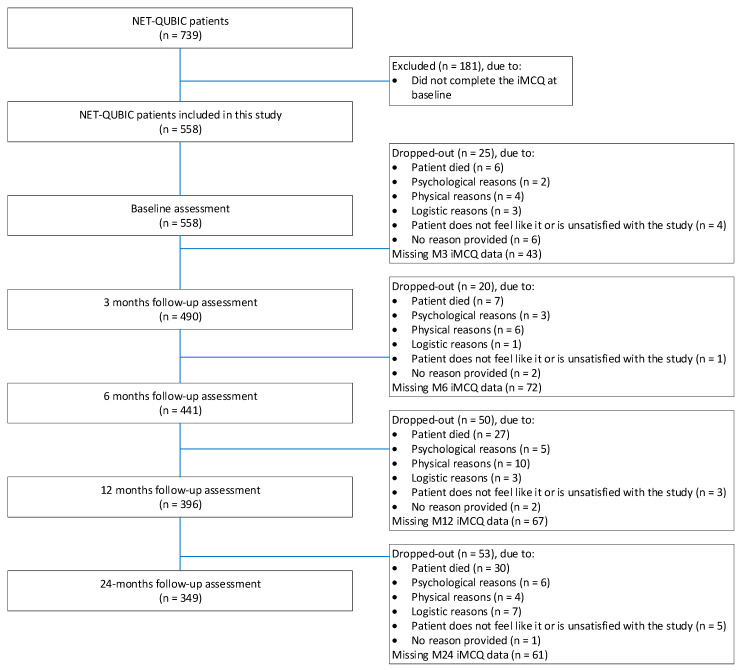
Medical consumption questionnaire (iMCQ).

The characteristics of the study population are shown in Table 1. The majority were male (74%) and the mean age was 64 years (range 19–86 years). Most patients had a stage III–IV tumor (57%). The tumors were most often located in the oropharynx (36%), followed by an oral cavity (28%), larynx (27%), hypopharynx (6%), and unknown primary (3%). Approximately one third of the patients (33%) were treated with radiotherapy, 21% of the patients were treated with surgery, and 45% of the patients were treated with a combination of treatment modalities (chemoradiation, or surgery and (chemo)radiotherapy). In total, 88% of all 558 patients included in this study completed at least one follow-up measure (Figure 1). Reasons for drop-out are shown in Figure 1. More detailed information on the study flow is provided in a previous published study [30]. 

The prevalence rate of a high level of distress was 33% at baseline, and 17% at 12-month follow-up. The prevalence rate of symptoms of anxiety was 26% at baseline and 9% at 12-month follow-up, and of symptoms of depression was 14% at baseline and 9% at 12-month follow-up. FCR was found in 37% of the patients at baseline and 29% at 12-month follow-up. The prevalence rates of anxiety and depression disorders were substantially lower; 2% and 3% at baseline and 1% and 6% at 12-month follow-up, respectively. 

### 3.2. Use of Healthcare and Informal Care

Frequencies of healthcare and informal care use are presented in Table 2. Use of mental healthcare was relatively low at all time points (<9%). Use of primary care ranged from 92% at baseline to 57–59% at 12- and 24-month follow-up. Use of supportive care ranged from 54–80%, of which physical therapy, dietician care, and oral hygiene care were used most often, and social work and support groups were used less often (<5%). The use of informal care ranged from 9–24%. 

Patients who used mental healthcare and primary care reported, on average, three to four visits in the previous 3 months. Patients who used supportive care reported, on average, four to nine visits, and patients who used informal care received, on average, between 29 and 58 h of care in the last 3 months. 

### 3.3. Psychological Problems in Relation to Use of Healthcare and Informal Care

Results of univariate analyses testing psychological problems in relation to healthcare and informal care use are shown in Appendix A and Appendix B. Psychological problems that were significantly associated with care use in the univariate models were further analyzed in multivariate analyses with correction for potential confounders (Table 3); this excludes the associations with mental healthcare use, as a consequence of low mental healthcare use. Distress, symptoms of anxiety, symptoms of depression, FCR, anxiety disorder, and depression disorder were significantly associated with higher mental healthcare use for at least one time point. Use of mental healthcare ranged from 3% to 7% among patients without psychological problems, and from 6% to 50% among patients with psychological problems (Appendix A and Appendix B). Anxiety disorder at 12-month follow-up in relation to care use could not be analyzed due to an insufficient sample size (i.e., <10 patients with an anxiety disorder at 12-month follow-up).

### 3.4. Psychological Problems in Relation to Costs

Results of the analyses regarding associations between psychological problems at baseline, and costs at baseline, 3-, 6-, 12-, and 24-month follow-up, adjusted for sociodemographic and clinical factors, are shown in Table 4. Psychological distress, symptoms of anxiety, and FCR at baseline were significantly associated with higher costs in the 3 months before baseline assessment (probability >98.3%). Patients with distress at baseline had, on average, €93 (BCa 95% CI = €18; €180) higher costs at baseline, patients with symptoms of anxiety at baseline had, on average, €125 (BCa 95% CI = €45; €231) higher costs at baseline, and patients with FCR at baseline had, on average, €80 (BCa 95% CI = €10; €162) higher costs at baseline, compared to patients without these psychological problems. 

Results of the analyses for the associations between psychological problems at 12-month follow-up, and costs at 12- and 24-month follow-up, adjusted for sociodemographic and clinical factors, are shown in Table 4. None of the associations were statistically significant. However, the probability approach showed that the probability that anxiety disorder at baseline was associated with higher costs at 6-month follow-up was high (probability of 89.3%). Furthermore, the probability that depression disorder at baseline was associated with higher costs at 3-, 6-, and 12-month follow-up, and that depression disorder at 12-month follow-up was associated with higher costs at 24-month follow-up, was high (probability between 89.6% and 95.1%). On the other hand, the probability that depression disorder at baseline was associated with higher costs at 24-month follow-up was low (probability of 5.2%).

## 4. Discussion

The aim of this study was to investigate the relationship between psychological problems and use of care and costs from baseline up to 2 years after treatment among HNC patients. Overall, the results of this study support the suggestion posed by Carlson and Bultz [13] that cancer patients with psychological problems not only make more use of mental healthcare, but also other types of healthcare. The results are also in line with the general findings of a systematic review that cancer patients with psychological problems make more use of mental and primary healthcare, and have higher healthcare costs [14]. 

In this exploratory prospective study, we specifically investigated the relationship between various types of psychological problems (distress, symptoms of anxiety and depression, FCR, and anxiety and depression disorder) and various types of care (mental, primary, supportive, and informal care) and costs. We found that HNC patients with distress, symptoms of anxiety, or FCR at time of diagnosis had significantly more costs in the 3 months prior to diagnosis. It was also highly likely that patients with psychological problems at baseline had more costs compared to patients without psychological problems at 3- (depression disorder), 6- (anxiety and depression disorder), and 12- (depression disorder) month follow-up, and that patients with psychological problems 12 months after treatment had higher costs at 12- (distress) and 24- (depression disorder) month follow-up. In addition, patients with symptoms of anxiety at baseline made more use of primary care and supportive care, and patients with FCR or an anxiety disorder used informal care more often, 3 months after treatment. Patients with symptoms of depression at baseline made more use of informal care 6 months after treatment, and patients who had distress, symptoms of anxiety, or FCR more often made use of primary care at 12-month follow-up. Patients with symptoms of anxiety at 12-month follow-up made more use of primary care at that assessment time. Two years after treatment, patients with psychological problems at baseline or 12-month follow-up did not seem to make use of primary care as often, but made more use of supportive care (patients with distress or symptoms of anxiety at baseline, and patients with FCR at 12-month follow-up) and informal care (patients with a depression disorder at 12-month follow-up). 

The costs among patients with psychological problems before treatment and/or 12 months after treatment were, when likely to be more expensive (i.e., probability >89%), on average between €80 and €391 higher during a 3-month time period compared to patients without these psychological problems. Other studies reported that cancer patients with a depression disorder or anxiety disorder had, on average, between $6000 to $25,000 and $15,000 to $60,000 higher costs, respectively, in a year [17,31,32,33]. A reason for this cost difference might be that, in our study, only costs related to mental, primary, supportive and informal care were included, whereas in these other studies, additional costs of inpatient, outpatient, and emergency room visits were included. Another explanation may be that these other studies focused on psychiatric disorders, and increased care use and costs are especially prevalent among those with psychiatric disorders. 

An explanation for the higher costs among patients with psychological symptoms or disorders may be that patients with a poorer clinical status (comorbidity, more advanced cancer stage) are more likely to develop psychological symptoms or a psychiatric disorder [7,34]. Therefore, higher healthcare use among those with psychological symptoms/disorders might not be a result of psychological symptoms/disorder, but instead be a result of a poorer clinical status. To account for this, we adjusted for confounders at baseline, such as cancer stage, treatment modalities, and comorbidity. Although some associations were no longer significant after adjustment, several associations remained significant, indicating that associations between psychological problems and healthcare use and costs do not (entirely) result from worse clinical outcomes. 

Other explanations for higher care use and costs among those with psychological symptoms or disorders, as previously hypothesized by Carlson and Bultz [13], are that patients with psychological problems may be less likely to fully adhere to medical treatment, and that they are less likely to maintain a healthy lifestyle; these factors may lead to decreased overall health at follow-up, and, consequently, an increased need for and use of healthcare services. Surprisingly, results at 24-month follow-up suggest that patients with psychological problems at baseline have lower costs. An explanation for this may be that the association between psychological problems and costs is especially present at short-term follow-up, and that later on, other factors become more important in the association with costs. In the current study, it seems that patients with a psychiatric disorder made solely more use of informal care (and not professional primary or supportive care), whereas patients with distress, symptoms of anxiety or depression, or FCR made more use of professional care (i.e., primary care and supportive care). Due to limited power, the use of mental healthcare could not be investigated via multivariate models. However, univariate analyses showed that mental healthcare was used relatively more often by patients with a psychiatric disorder (mental health care was used by up to 40% of patients with a disorder at baseline versus 16% among patients with psychological symptoms). This suggests that patients with a psychiatric disorder are more likely to be referred to mental healthcare, whereas patients with psychological symptoms may be more likely to be referred to supportive care, or to consult their general practitioner. Another observation is that, seemingly, patients with symptoms of depression, or a depression disorder, made more use of informal care, whereas patients with symptoms of anxiety, FCR, or an anxiety disorder, made more use of both primary care and informal care. An explanation for this may be that anxious patients visit healthcare providers more often to be reassured that their health is under control. 

To unravel these potential differences, further research is needed on the course of healthcare utilization after diagnosis, and the moderation or mediating effects of psychological problems, cancer recurrence, lifestyle behavior, and treatment adherence. The use of mental healthcare in this study population was low, which may be related to suboptimal organization of care and/or willingness to accept mental healthcare by patients. Brebach et al. [35] estimated that 60% of cancer patients with distress, anxiety, or depression accept psychological treatment when offered [35]. This percentage was also reported in a recent study among mixed cancer patients with adjustment disorders [36]. Further research is needed on factors that may explain why some patients receive psychological care in clinical practice and some do not, including the role of the patient him/herself (e.g., coping style, a self-perceived need for psychological care). 

A key strength of this study is the longitudinal design, which enabled prospective analyses of associations between psychological problems and care use and costs. Another strength of this study is that patient-reported outcomes and diagnostic interviews were used to identify patients with psychological symptoms and patients with an anxiety or depression disorder, respectively. Previous studies used health insurance data [17,18] derived from routine care; however, it is known that psychological symptoms and psychiatric disorders often remain undiagnosed among cancer patients [37]. This may have resulted in an underestimation of the cost difference. Additionally, in the current study, we controlled for sociodemographic and clinical confounders. A potential limitation of this study is the large number of analyses performed. We did not perform a Bonferroni assessment, as different psychological problems were investigated in relation to different types of healthcare use [38]. Moreover, sample size was too small to conduct multivariate analyses with respect to mental healthcare use. The small number of patients with an anxiety or depression disorder necessitates caution in interpreting the results. Furthermore, it would be interesting to include medication use in the analysis. Unfortunately, these data are not yet available.

Finally, this study investigated the associations between psychological problems and care use and costs among Dutch cancer patients. Use of care may be limited or driven by health insurance systems [39]. 

## 5. Conclusions

HNC patients with psychological problems more often use healthcare and informal care, and have higher costs. This association remained after adjusting for demographic and clinical characteristics, indicating that the association does not result from worse clinical outcomes in patients with psychological problems.

## Figures and Tables

**Table 1:** Characteristics of included and excluded patients.

	Excluded Patients (n = 181)	Included Patients ^2^ (n = 558)	*p*-Value
Mean age (SD)	62 (11)	64 (9)	0.07
Women	43 (24%)	147 (26%)	0.49
Living alone	57 (43%)	106 (21%)	<0.01
Education level ^1^			
Low	64 (48%)	215 (42%)	0.13
Middle	37 (28%)	134 (26%)	
High	31 (24%)	167 (32%)	
Tumor site			
Oral cavity	43 (24%)	156 (28%)	0.36
Oropharynx	63 (35%)	199 (36%)	
HPV positive	26 (41%)	104 (52%)	
HPV negative	30 (48%)	69 (35%)	
HPV unknown	7 (11%)	26 (13%)	
Hypopharynx	18 (10%)	34 (6%)	
Larynx	53 (29%)	152 (27%)	
Unknown primary	4 (2%)	17 (3%)	
Clinical tumor stage ^3^			
0/I/II	57 (31%)	238 (43%)	0.01
III/IV	124 (69%)	320 (57%)	
Treatment			
Surgery	36 (20%)	116 (21%)	0.37
Radiotherapy	56 (31%)	185 (33%)	
Chemoradiotherapy	62 (34%)	153 (27%)	
Surgery and radiotherapy	20 (11%)	86 (15%)	
Surgery and chemoradiotherapy	6 (3%)	17 (3%)	
WHO performance status			
0	112 (62%)	395 (71%)	0.03
1 or more	69 (38%)	163 (29%)	
ACE-27 comorbidity			
None/mild	97 (58%)	371 (70%)	<0.01
Moderate/severe	71 (42%)	160 (30%)	
Psychological outcomes at baseline			
High level of distress (HADS-T ≥ 11)		206 (37%)	
Symptoms of anxiety (HADS-A ≥ 8)		146 (26%)	
Symptoms of depression (HADS-D ≥ 8)		80 (14%)	
High level of fear of recurrence (CWS ≥ 14)		251 (46%)	
Anxiety disorder		11 (2%)	
Depression disorder		14 (3%)	

^1^ Low education level includes primary education, lower or preparatory vocational education, and intermediary general secondary education. Middle education level includes senior general secondary education and higher general secondary education. High education level includes higher professional education and university. ^2^ There were 41 missing values on living status, 42 missing values on education level, 1 missing value on treatment, 27 missing values on comorbidity, 4 missing values on increased distress, 4 missing values on increased anxiety, 2 missing values on increased depression, 14 missing values on fear of cancer recurrence, 108 missing values on anxiety disorder, 109 missing values on depression disorder. ^3^ One patient had a clinical TNM of 0 and a pathological TNM of II, and was therefore included in the NET-QUBIC study. Abbreviations: HADS, Hospital Anxiety and Depression Scale; T, total; D, depression; A, anxiety; HPV, human papilloma virus; CWS, Cancer Worry Scale. All results are presented in N (%), unless otherwise stated.

**Table 2:** Use of care at baseline, 3-, 6-, 12-, and 24-month follow-up.

	Baseline (N = 558)		3-Month Follow-Up (N = 490)		6-Month Follow-Up (N = 441)		12-Month Follow-Up (N = 396)		24-Month Follow-Up (N = 349)	
	% Patients Using Service	Mean Number (SD) of Contacts or Hours *	% Patients Using Service	Mean Number (SD) of Contacts or Hours *	% Patients Using Service	Mean Number (SD) of Contacts or Hours *	% Patients Using Service	Mean Number (SD) of Contacts or Hours *	% Patients Using Service	Mean Number (SD) of Contacts or Hours *
Mental healthcare	3%	4.4 (6.4)	8%	2.9 (2.7)	8%	4.1 (3.5)	8%	3.0 (2.8)	4%	4.1 (3.8)
Primary care	92%	3.8 (3.3)	76%	4.2 (4.3)	66%	3.6 (4.5)	57%	3.3 (2.6)	59%	4.5 (20.7)
Supportive care	58%	4.3 (6.2)	80%	7.8 (9.2)	71%	9.0 (12.4)	60%	7.7 (11.5)	54%	7.06 (12.2)
Social work	4%	3.5 (4.7)	4%	6.8 (5.7)	5%	1.7 (1.6)	4%	3.4 (4.7)	4%	10.1 (12.1)
Physical therapy	13%	8.3 (8.0)	22%	8.1 (6.3)	32%	11.7 (10.9)	26%	10.4 (10.4)	25%	6.2 (13.0)
Support group	1%	12.3 (8.2)	4%	6.9 (5.8)	5%	19.4 (15.8)	4%	14.8 (11.4)	2%	10.75 (10.7)
Dietitian	27%	1.4 (1.3)	56%	3.0 (3.5)	27%	1.9 (1.6)	12%	2.0 (1.7)	8%	1.6 (0.9)
Speech therapy	9%	2.1 (2.1)	18%	2.8 (5.6)	16%	3.3 (3.8)	11%	4.9 (6.1)	6%	5.7 (6.7)
Oral care	38%	1.3 (0.7)	52%	3.5 (6.1)	39%	1.5 (1.2)	34%	1.3 (0.9)	28%	1.2 (1.0)
Informal care	13%	29.3 (39.4)	24%	61.0 (97.2)	17%	46.9 (90.4)	13%	49.2 (63.9)	9%	57.6 (74.7)

* Informal care and support group were measured in hours. Healthcare use and informal care were measured with a recall period of 3 months.

**Table 3:** Multivariate analyses testing associations between psychological problems at baseline and 12- months follow-up and use of care at baseline, 3-,6-,12-, and 24-month follow-up.

		Baseline (N = 558)		3-Month Follow-Up (N = 490)		6-Month Follow-Up (N = 441)		12-Month Follow-Up (N = 396)		24-Month Follow-Up (N = 396)	
		OR (95% CI)	*p*	OR (95% CI)	*p*	OR (95% CI)	*p*	OR (95% CI)	*p*	OR (95% CI)	*p*
Measured at baseline											
Distress	Mental healthcare			N/A		N/A				N/A	
	Primary care							1.74 (1.13–2.67)	0.01		
	Supportive care	1.59 (1.09–2.32)	0.02							1.78 (1.11–2.87)	**0.02**
	Informal care	1.48 (0.86–2.53)	0.16	1.36 (0.85–2.18)	0.20						
Symptoms of anxiety	Mental healthcare			N/A		N/A					
	Primary care	2.54 (1.05–6.11)	0.04			1.61 (0.99–2.62)	0.057	1.77 (1.10–2.87)	0.02		
	Supportive care	1.55 (1.02–2.36)	0.04	1.98 (1.07–3.67)	0.03					1.99 (1.17–3.36)	**0.01**
	Informal care			1.57 (0.94–2.60)	0.08	1.27 (0.70–2.31)	0.440				
Symptoms of depression	Mental healthcare					N/A					
	Primary care									1.54 (0.75–3.16)	0.24
	Supportive care										
	Informal care	1.68 (0.87–3.23)	0.12	1.62 (0.89–2.95)	0.12	2.58 (1.34–4.97)	0.005				
Fear of cancer recurrence	Mental healthcare			N/A		N/A					
	Primary care							1.60 (1.06–2.40)	0.03		
	Supportive care			1.64 (1.00–2.70)	0.05					1.47 (0.94–2.29)	0.09
	Informal care			1.71 (1.06–2.76)	0.03						
Anxiety disorder	Mental healthcare					N/A					
	Primary care										
	Supportive care										
	Informal care			4.76 (1.04–21.81)	0.045						
Depression disorder	Mental healthcare	N/A		N/A		N/A					
	Primary care										
	Supportive care										
	Informal care										
Measured at 12-months follow-up											
Distress	Mental healthcare							N/A			
	Primary care										
	Supportive care										
	Informal care							2.08 (0.88–4.92)	0.10		
Symptoms of anxiety	Mental healthcare							N/A		N/A	
	Primary care							2.71 (1.11-6.62)	0.03		
	Supportive care										
	Informal care									2.90 (0.84-10.06)	0.09
Symptoms of depression	Mental healthcare										
	Primary care										
	Supportive care										
	Informal care										
Fear of cancer recurrence	Mental healthcare										
	Primary care										
	Supportive care									1.74 (1.01-2.98)	**0.04**
	Informal care										
Depression disorder	Mental healthcare							N/A		N/A	
	Primary care										
	Supportive care										
	Informal care									6.42 (1.64-9.81)	**0.01**

Abbreviations: N/A, not applicable due to insufficient sample size; CI, confidence interval; OR, odds ratio. Odds ratios are adjusted for age, sex, living status, education level, tumor site, tumor stage, treatment, performance state, comorbidity. Bold represents significance (*p* < 0.05). With regard to associations between psychological problems and primary, supportive, and informal care use, multivariate analyses showed that symptoms of distress at baseline were significantly associated with use of primary care at 12-month follow-up (OR =1.74, 95%CI = 1.13–2.67) and use of supportive care at baseline (OR = 1.59, 95% CI = 1.09–2.32) and 24-month follow-up (OR = 1.78, 95% CI = 1.11–2.87). Symptoms of anxiety were significantly associated with use of primary care at baseline (OR = 2.54, 95% CI = 1.05–6.11) and 12-month follow-up (OR = 1.77, 95% CI = 1.10–2.87), and use of supportive care at baseline (OR = 1.55, 95% CI = 1.02–2.36), 3-month follow-up (OR = 1.98, 95% CI = 1.07–3.67), and 24-month follow-up (OR = 1.99, 95% CI = 1.17–3.61). Symptoms of depression were significantly associated with use of informal care at 6-month follow-up (OR = 2.58, 95% CI = 1.34–4.97). Symptoms of FCR were significantly associated with use of primary care at 12-month follow-up (OR = 1.60, 95% CI = 1.06–2.40) and use of informal care at 3-month follow-up (OR = 1.71, 95% CI = 1.06–2.76). Anxiety disorder was significantly associated with use of informal care at 3-month follow-up (OR = 4.76, 95% CI = 1.04–21.81). With regard to psychological problems, multivariate analysis showed that symptoms of anxiety were significantly associated with use of primary care at 12-month follow-up (OR = 2.71, 95% CI = 1.11–6.62). FCR was associated with use of supportive care at 24-month follow-up (OR = 1.74, 95% CI = 1.01–2.98). Depression disorder was associated with informal care use at 24-month follow-up (OR = 6.42, 95% CI = 1.64–9.81).

**Table 4:** Differences in costs at baseline, 3-,6-,12-, and 24-month follow-up between patients with and without psychological problems at baseline and 12-months follow-up.

	Baseline		3-Month Follow-Up		6-Month Follow-Up		12-Month Follow-Up		24-Month Follow-Up	
	Mean (95% BCa CI)	% *	Mean (95% BCa CI)	% *	Mean (95% BCa CI)	% *	Mean (95% BCa CI)	% *	Mean (95% BCa CI)	% *
Measured at baseline										
Distress	**€93 (18; 180)**	**99.1%**	€21 (−230; 214)	58.7%	€−58 (−283; 90)	28.2%	€−5 (−158; 141)	48.7%	€100 (−105; 534)	74.1%
Symptoms of anxiety	**€125 (45; 231)**	**99.7%**	€−40 (−252; 203)	34.5%	€−83 (−291; 81)	19.1%	€−15 (−166; 149)	42.6%	€−12 (−248; 182)	45.3%
Symptoms of depression	€49 (−79; 215)	76.1%	€24 (−203; 311)	56.3%	€−85 (268; 145)	20.3%	€−30 (−194; 196)	36.1%	€−104 (−328; 168)	19.9%
Fear of cancer recurrence	**€80 (10; 162)**	**98.3%**	€−66 (−273; 98)	24.5%	€−18 (−194; 127)	42.6%	€94 (−58; 276)	87.8%	€4 (−185; 141)	52.4%
Anxiety disorder	€−68 (−277; 141)	26.1%	€393 (−301; 2706)	69.1%	€418 (−128; 1391)	89.3%	€262 (−149; 1086)	82.1%	€−167 (−847; 177)	23.6%
Depression disorder	€89 (−156; 646)	68.2%	€547 (−41; 2077)	91.1%	€388 (−108; 1238)	89.9%	€422 (−21; 1045)	95.1%	€−284 (−705; 36)	5.2%
Measured at 12-months follow-up										
Distress							€166 (−58; 477)	89.6%	€−33 (−247; 215)	37.0%
Symptoms of anxiety							€42 (−161; 406)	60.8%	€124 (−167; 570)	75.1%
Symptoms of depression							€165 (−85; 660)	82.7%	€20 (−196; 574)	51.0%
Fear of cancer reoccurrence							€89 (−120; 498)	72.0%	€67 (−118; 236)	76.3%
Depression disorder							€−81 (−286; 258)	26.1%	€391 (−188; 1061)	89.6%

Abbreviations: BCa CI, bias-corrected and accelerated bootstrap confidence intervals. * Probability that the group with psychological problems had higher costs. Results are adjusted for age, sex, living status, education level, tumor site, tumor stage, treatment, performance state, comorbidity. Bold represents significance (*p* < 0.05).

## Data Availability

The datasets generated and analyzed during the current study are not publicly available as the collection and integration of large amounts of personal, biological, genetic and diagnostic information precludes open access to the NET-QUBIC research data. Data are available from the corresponding author on reasonable request. On the NET-QUBIC website (www.kubusproject.nl) is described how NET-QUBIC data are made available for the research community.

## Appendix A

**Table A1:** Psychological problems at baseline in relation to healthcare use at baseline, 3-, 6-, 12-, and 24-month follow-up.

	Distress		Symptoms of Anxiety		Symptoms of Depression		Fear of Cancer Recurrence		Anxiety Disorder		Depression Disorder	
	Yes	No	Yes	No	Yes	No	Yes	No	Yes	No	Yes	No
	% Patients Using Service		% Patients Using Service		% Patients Using Service		% Patients Using Service		% Patients Using Service		% Patients Using Service	
**Baseline (N = 558)**	(N = 206)	(N = 408)	(N = 146)	(N = 408)	(N = 80)	(N = 476)	(N = 251)	(N = 307)	(N = 11)	(N = 439)	(N = 14)	(N = 435)
Mental healthcare	5%	2%	5.5%	3%	6%	3%	4%	3%	9%	3%	**21%**	**3%**
General practitioner	93%	91%	**96%**	**90%**	92%	92%	94%	90%	91%	92%	100%	92%
Other supportive care	**66%**	**53%**	**67%**	**54%**	56%	58%	62%	55%	45%	56%	36%	57%
Informal care	**17%**	**10%**	16%	12%	**21%**	**11%**	14%	11%	18%	12%	0%	12%
**3-month follow-up (N = 490)**	(N = 173)	(N = 314)	(N = 124)	(N = 363)	(N = 65)	(N = 423)	(N = 220)	(N = 256)	(N = 8)	(N = 396)	(N = 10)	(N = 393)
Mental healthcare	**13%**	**6%**	**16%**	**6%**	14%	8%	**11%**	**6%**	25%	7%	**30%**	**7%**
General practitioner	77%	76%	77%	76%	83%	75%	79%	73%	100%	75%	90%	75%
Other supportive care	81%	79%	**87%**	**76%**	74%	81%	**86%**	**75%**	100%	79%	80%	80%
Informal care	**30%**	**21%**	**32%**	**21%**	**37%**	**22%**	**28%**	**20%**	**63%**	**23%**	30%	24%
**6-month follow-up (N = 441)**	(N = 160)	(N = 278)	(N = 117)	(N = 321)	(N = 62)	(N = 377)	(N = 196)	(N = 233)	(N = 6)	(N = 355)	(N = 10)	(N = 351)
Mental healthcare	**11%**	**5%**	**14%**	**5%**	**15%**	**6%**	**11%**	**5%**	**33%**	**7%**	**40%**	**6%**
General practitioner	71%	63%	**74%**	**63%**	68%	66%	69%	64%	100%	66%	70%	66%
Other supportive care	71%	70%	74%	69%	69%	71%	75%	67%	83%	71%	70%	71%
Informal care	23%	13%	**23%**	**15%**	**36%**	**14%**	20%	14%	33%	16%	100%	16%
**12-month follow-up (N = 396)**	(N = 136)	(N = 257)	(N = 97)	(N = 296)	(N = 54)	(N = 340)	(N = 173)	(N = 213)	(N = 6)	(N = 316)	(N = 8)	(N = 314)
Mental healthcare	11%	6%	12%	6%	9%	8%	10%	5%	0%	6%	13%	5%
General practitioner	**65%**	**52%**	**67%**	**53%**	63%	56%	**63%**	**52%**	83%	56%	63%	56%
Other supportive care	61%	60%	61%	60%	56%	61%	61%	59%	83%	59%	50%	60%
Informal care	14%	12%	14%	12%	20%	12%	15%	11%	0%	11%	0%	12%
**24-month follow-up (N = 349)**	(N = 114)	(N = 232)	(N = 82)	(N = 264)	(N = 40)	(N = 307)	(N = 151)	(N = 191)	(N = 5)	(N = 283)	(N = 7)	(N = 281)
Mental healthcare	**6%**	**3%**	7%	3%	5%	4%	5%	3%	20%	4%	0%	4%
General practitioner	63%	57%	66%	57%	55%	60%	58%	60%	100%	60%	57%	61%
Other supportive care	**65%**	**48%**	**67%**	**49%**	**65%**	**52%**	**60%**	**48%**	80%	51%	43%	52%
Informal care	12%	8%	15%	8%	10%	9%	10%	8%	0%	8%	0%	8%

Bold represents significance (*p* < 0.05).

## Appendix B

**Table A2:** Psychological problems at 12-month follow-up in relation to healthcare use at baseline, 12-, and 24-month follow-up.

	Distress		Symptoms of Anxiety		Symptoms of Depression		Fear of Cancer Recurrence		Depression Disorder	
	Yes	No	Yes	No	Yes	No	Yes	No	Yes	No
	% Patients Using Service		% Patients Using Service		% Patients Using Service		% Patients Using Service		% Patients Using Service	
**12-month follow-up (N = 396)**	(N = 54)	(N = 263)	(N = 29)	(N = 289)	(N = 29)	(N = 292)	(N = 98)	(N = 244)	(N = 22)	(N = 301)
Mental healthcare	**20%**	**4%**	**31%**	**5%**	17%	6%	11%	5%	**18%**	**6%**
General practitioner	65%	53%	**76%**	**53%**	69%	54%	59%	55%	59%	56%
Other supportive care	63%	60%	66%	60%	69%	60%	62%	59%	64%	60%
Informal care	**22%**	**10%**	17%	11%	24%	11%	14%	12%	14%	10%
**24-month follow-up (N = 349)**	(N = 40)	(N = 233)	(N = 22)	(N = 252)	(N = 19)	(N = 257)	(N = 81)	(N = 212)	(N = 16)	(N = 281)
Mental healthcare	8%	3%	**14%**	**3%**	11%	4%	7%	3%	**19%**	**3%**
General practitioner	70%	58%	73%	59%	74%	59%	63%	58%	63%	61%
Other supportive care	58%	51%	64%	51%	58%	52%	**64%**	**49%**	56%	51%
Informal care	13%	8%	**23%**	**8%**	11%	9%	7%	9%	**31%**	**8%**

Bold represents significance (*p* < 0.05).
